# The involvement of mast cells in the irinotecan-induced enteric neurons loss and reactive gliosis

**DOI:** 10.1186/s12974-017-0854-1

**Published:** 2017-04-07

**Authors:** Ludmila T. Nogueira, Deiziane V. S. Costa, Antoniella S. Gomes, Conceição S. Martins, Angeline M. H. P. Silva, Juliana M. Coelho-Aguiar, Patrícia Castelucci, Roberto C. P. Lima-Júnior, Renata F. C. Leitão, Vivaldo Moura-Neto, Gerly A. C. Brito

**Affiliations:** 1 Department of Morphology, Federal University of Piauí, Teresina, Piauí Brazil; 2 Department of Morphology, Faculty of Medicine, Federal University of Ceará, Farias Delmiro street, Fortaleza, CE 60430170 Brazil; 3 Paulo Niemeyer Brain Institute, Federal University of Rio de Janeiro, UFRJ, Rio de Janeiro, RJ Brazil; 4 Department of Anatomy, University of São Paulo, São Paulo, SP Brazil; 5 Department of Physiology and Pharmacology, Faculty of Medicine, Federal University of Ceará, Fortaleza, Ceará Brazil

**Keywords:** Irinotecan, Mucositis, Enteric nervous system, Mast cell, Glia, Neuron

## Abstract

**Background:**

The irinotecan (CPT-11) causes intestinal mucositis and diarrhea that may be related to changes in the enteric nervous system (ENS). In inflammatory condition, mast cells release a variety of pro-inflammatory mediators that can interact with the ENS cells. It has not been explored whether CPT-11 is able to alter the enteric glial and neuronal cell, and the role of mast cells in this effect. Therefore, this study was conducted to investigate the effect of CPT-11 on the enteric glial and neuronal cells, as well as to study the role of mast cells in the CPT-11-induced intestinal mucositis.

**Methods:**

Intestinal mucositis was induced in Swiss mice by the injection of CPT-11 (60 mg/kg, i.p.) once a day for 4 days following by euthanasia on the fifth day. To investigate the role of mast cells, the mice were pretreated with compound 48/80 for 4 days (first day, 0.6 mg/kg; second day, 1.0 mg/kg; third day, 1.2 mg/kg; fourth day, 2.4 mg/kg) to induce mast cell degranulation before the CPT-11 treatment.

**Results:**

Here, we show that CPT-11 increased glial fibrillary acidic protein (GFAP) and S100β gene and S100β protein expressions and decreased HuC/D protein expression in the small intestine segments. Concomitantly, CPT-11 enhanced tumor necrosis factor-α (TNF-α) and interleukin-6 (IL-6) levels and inducible nitric oxide synthase (iNOS) gene expression, associated with an increase in the total number macrophages (positive cells for ionized calcium-binding adapter molecule, Iba-1) and degranulated mast cells in the small intestine segments and caused significant weight loss. The pretreatment with compound 48/80, an inductor of mast cells degranulation, significantly prevented these CPT-11-induced effects.

**Conclusions:**

Our data suggests the participation of mast cells on the CPT-11-induced intestinal mucositis, macrophages activation, enteric reactive gliosis, and neuron loss.

**Electronic supplementary material:**

The online version of this article (doi:10.1186/s12974-017-0854-1) contains supplementary material, which is available to authorized users.

## Background

Irinotecan (CPT-11) is a topoisomerase I inhibitor, broadly used to treat lung, gastric, cervical, ovarian, and colorectal cancers [1]. The active metabolite, SN-38, induces irreversible deoxyribose nucleic acid (DNA) damage in tumor cells, and its accumulation in the intestinal mucosa is thought to be responsible for enterotoxicity [2]. Irinotecan-based cancer chemotherapy regimens induce intestinal mucositis in 25% of patients, which exhibit severe mucosal inflammation and life-threatening diarrhea. [3, 4] There has been increasing interest in the pathobiology of antineoplastic-induced intestinal mucositis, since it compromises the treatment outcome and may increase morbidity and mortality [5].

Previous reports demonstrated that patients using chemotherapeutic drugs, such as CPT-11, present gastrointestinal complications, such as intestinal dysmotility [6]. Delayed gastric empty and increased intestinal contractility were associated with 5-Fluorouracil-induced intestinal mucositis in mice [7]. The involvement of many inflammatory mediators, such as nitric oxide (NO) [8], interleukin-18 (IL-18) [9], interleukin-33 (IL-33) [10], tumor necrosis factor-α (TNF-α), interleukin-1β (IL-1β), and keratinocyte chemoattractant (KC) [11] have been implicated on these side effects, leading to intestinal damage, characterized by shortened villi, loss of crypts architecture, and intense inflammatory cell infiltrate. The consequences of irinotecan in the enteric nervous system (ENS), a ganglionated neuronal network that resides within the gut wall, however, have not been studied and merit further investigation.

Pronounced reduction of total neuron number, associated with an increase in the number of enteric glia cells (EGC) in ENS is reported in several bowel inflammatory disorders, such as celiac disease [12], inflamed segments of Crohn’s diseases [13], and ulcerative colitis [14–17]. Crohn’s disease [18] and irritable bowel syndrome [19] are both characterized by intense inflammation and by augmented mast cell infiltration. Mast cell mediators release, such as histamine, tryptase, prostaglandin D2 (PGD2) and interleukin-6 (IL-6) stimulate intestinal secretion and motility [20, 21]. Furthermore, mast cells mediators were already implicated in neuronal death [22].

In this study, we investigated whether ENS is affected by CPT-11-induced intestinal mucositis, and the potential participation of mast cells in this effect. We demonstrated that CPT-11 enhanced and activated EGCs, reduced the neuron population, and augmented the number and activation of mast cells in the small intestine. These effects were prevented by previous degranulation of mast cells.

## Methods

### Animals

Seventy-two Swiss mice, weighing 25–30 g, were housed in temperature-controlled rooms under 12-h light-dark cycles. The animals received water and food ad libitum. The animal treatments were conducted in accordance with the Guidelines for Institutional and Animal Care and Use of Federal University of Ceará, Brazil.

All procedures involving animals were approved by the Federal University of Ceará Committee on the ethical treatment of research animals (protocol no. 84/2015).

### Induction of experimental intestinal mucositis

Intestinal mucositis was induced in Swiss mice, as previously described [23], by four injections, once daily, of irinotecan (CPT-11, 60 mg/kg, i.p.). The animals were euthanized with an overdose of 2% xylazine and 10% ketamine (10/100 mg/kg; i.p.) 5 days after the first CPT-11 administration. The body weight was monitored throughout the experimental periods.

### Experimental groups

The mice were randomly divided into four groups: (1) the control group received intraperitoneal 0.9% saline solution once daily for 4 days; (2) the CPT-11 group received irinotecan (CPT-11, 60 mg/kg, i.p.) once daily for 4 days; (3) the CPT-11 + c48/80 group was pretreated for 4 days with the mast cell degranulator compound 48/80 (Sigma-Aldrich) dissolved in PBS (phosphate-buffered saline; injected with 200 μl/cavity, i.p.), as previously described in the literature [24], according to the following schemes: 0.6 mg/kg on the first day; 1.0 mg/kg on the second day; 1.2 mg/kg on the third day, and 2.4 mg/kg on the fourth day. The induction of intestinal mucositis by CPT-11was initiated 24h after the last administration of compound 48/80, as previously described in the present work; (4) the c48/80 group was pretreated with compound 48/80, as described, but was not subjected to CPT-11-induced intestinal mucositis.

### Toluidine blue staining

Following euthanasia, 5 days after the first CPT-11 injection, tissue samples (including the mucosal, submucosal, muscle, and serosa layer) of each small intestine sections (duodenum, jejunum and ileum) were collected and fixed in 10% neutral-buffered formalin, dehydrated, and embedded in paraffin. Paraffin-embedded tissue sections were mounted onto slides, deparaffinized with xylene, incubated for 3 min with toluidine blue solution (1 g of toluidine blue dissolved in 70% ethanol), washed three times in distilled water, dehydrated, and mounted. Mast cell granules stain purple in color due to the presence of heparin and histamine. For that reason, purple stain was recognized as a positive staining for mast cells. The total number of mast cells (degranulated and non-degranulated) were counted manually, considering six specimens per group and ten fields per slide (where the highest number of mast cells was seen), on ×400 magnification. The results are expressed as the percentage of degranulated mast cells.

### Western blot analyses

Following euthanasia, 5 days after the first CPT-11 injection, samples of the three small intestine segments (duodenum, jejunum, and ileum) were collected and homogenized in RIPA lysis buffer (25 mM Tris-HCL, pH 7.6; 150 mM NaCl; 5 mM EDTA; 1% NP40; 1% triton X-100; 1% sodium deoxilato; 0,1% SDS) and protease inhibitor (1 μL inhibitor: 100 μL RIPA). For protein extraction, intestinal samples were centrifuged (17 min, 4 °C, 13,000 rpm), and supernatant was collected. Protein concentrations were determined through the bicinchroninic acid assay (Thermo Fisher Scientific) according to the manufacturer`s protocol. SDS-polyacrylamide gel electrophoresis (10%) was performed using 20 μg of protein (previously prepared with laemmli sample buffer, BioRad) and was denatured at 95 °C for 5 min. Thereafter, proteins were transferred to PVDF membrane (BioRad), blocked with BSA 5% for 1 h, and incubated overnight with primary antibody (rabbit anti-β actin, 04-1116, Merck Millipore, 1:500; goat anti-S100β, sc7851, Santa Cruz Biotechnology; or mouse anti-HuC/D, A21271, Invitrogen, 1 ug/mL) and secondary antibody (goat anti-rabbit, 656120, Invitrogen, 1:1000; rabbit anti-goat, A16142, Invitrogen, 1:2000; or goat anti-mouse IgG, 626520, Invitrogen, 1:1000). Membranes were incubated using the ECL system according to the manufacturer’s instructions (BioRad), and the chemiluminescence signal was detected using the ChemiDoc™ XRS system (BioRad). The densitometric quantification of western blot bands was quantified using NIH ImageJ software.

### Histopathological analysis

For the histological analysis, tissue samples of each small intestine segments were collected and fixed in 10% neutral-buffered formalin, dehydrated, and embedded in paraffin. Sections (5 μm thick) were obtained for hematoxylin-eosin staining (H&E) for subsequent examination using light microscopy (×200). The lengths of the intestinal villi (six to ten villi per slide; six mice per group) were determined from digital images captured by a digital camera coupled to a Leica microscope and were analyzed using ImageJ 1.4 software (NIH, Bethesda, MD, USA). Mucosa injuries were assessed using a modification of the histopathological score system described by Woo et al. [25] and were graded as follows: score 0, normal histological findings (no lesion); score 1, <10% crypts contain individual necrotic cells; score 2, >10% crypts contain necrotic cells, but the crypt architecture is intact; score 3, >10% crypts contain necrotic cells showing focal loss of crypt architecture, shortened villi and variable hypertrophy/hiperbasophilia apparent in the remaining crypt cells; and score 4, same as grade 3 except that the loss of crypt architecture and villus shortening are more extensive.

### Measurement of TNF-α and IL-6 in duodenum and jejunum

Duodenum and jejunum samples were collected 5 days after the first injection of CPT-11 for TNF-α and IL-6 measurements using a commercial ELISA kit (R&D System), according to the manufacturer’s instructions. The results are expressed as picograms per milliliter of tissue.

### Immunohistochemistry

Sections of 4 μm were prepared from paraffin-embedded intestinal tissues. After deparaffinization, antigenic recuperation was performed with citrate buffer (pH 6.0) for 20 min in 95 °C. Endogenous peroxidase was blocked with 3% H_2_O_2_ for 10 min to reduce non-specific binding. The sections were then incubated with glial fibrillary acidic protein (GFAP, IS524, DAKO), S100β (IR504, DAKO), HuC/D (A21271, Invitrogen, 1μg/mL), or ionized calcium-binding adapter molecule (Iba-1, ab107159, Abcam, 1:200) antibody, diluted in DAKO antibody diluent for 1 h. Sections were then incubated for 30 min with polymer (K4061, DAKO). The antibody binding sites were visualized by the incubation with diaminobenzidine–H_2_O_2_ (DAB, DAKO) solution. Sections incubated with antibody diluent, without the primary antibody included, were considered negative controls. Brownish color in the cytoplasm (GFAP, S100β, or HuC/D) or nucleus (HuC/D) was recognized as positive staining. The quantitative estimation of DAB products from immunostaining was determined from digital images of at least ten different areas of each section (from four specimens per group), where the highest number of immunostaining was detected, on ×400 (GFAP and S100β) or ×1000 (HuC/D) magnification, using Adobe Photoshop software. Quantification of the immunostaining was calculated dividing the number of positive pixels identified by DAB staining by the total number of pixels per image, as previously described [26]. The Iba-1 positive cells were manually counted from images of the small intestinal segments (duodenum, jejunum, and ileum), captured using a digital camera coupled to a Leica microscope, on ×400 magnification. It was considered four specimens per group, and ten fields per slide.

### RNA extraction and cDNA synthesis

The ribonucleic acid (RNA) was isolated from duodenum segments, collected 5 days after the first administration of CPT-11, using an established RNA isolation protocol, according to the manufacturer’s instructions (PROMEGA). RNA was quantified by NanoDrop (Thermo Fisher Scientific), and the samples purity was verified by 260/280 ratios >1.8. Complementary DNA (cDNA) was then synthesized from 1 μg RNA, using a High-Capacity cDNA Reverse Transcription Kit (Applied Biosystems), according to the manufacturer’s protocol.

### TaqMan qPCR

The messenger RNA (mRNA) expression analysis was performed by TaqMan quantitative PCR (qPCR), according to the manufacturer’s instruction, using pre-made probes (Applied Biosystems, USA), described in Table 1. All samples were run in duplicate, and the relative mRNA expression level was determined after normalizing all values to those of glyceraldehyde-3-phosphate dehydrogenase (Gapdh). For each sample, the following reagents were pipetted into a nuclease-free 0.2-mL microcentrifuge tube: 40 ng of cDNA (diluted ×5; 4 μl), 20x TaqMan gene expression assay (1 μl of S100β, GFAP, inducible nitric oxide synthase, iNOS or Gapdh), 2x TaqMan gene expression master mix (10 μl), and RNA-free water (5 μl). The reaction components were mixed by inverting the tube several times followed by a brief centrifugation. The 20 μl of PCR reaction mix (corresponded to the final volume of each reaction) were transferred into each well of a 96-well reaction plate. The plate was sealed and briefly centrifuged. The thermocycler parameters were set at 50 °C for 2 min, followed by 95 °C for 10 min, 40 cycles of 95 °C for 15 s, and 60 °C for 60 s. The fold changes were calculated using the ΔΔC_t_ comparative quantification method.

**Table 1:** Description of TaqMan probe used for the detection of target genes in the experiment

GFAP (glial fibrillary acidic protein)	
ID assay	Mm01253033_m1
TaQman probe	AGAAAACCGCATCACCATTCCTGTA
Amplicon length:	75
S100β (S100 protein, beta polypeptide, neural)	
ID assay	Mm00485897_m1
TaQman probe	CTTCCTGGAGGAAATCAAGGAGGAGCAG
Amplicon length:	69
iNOS (nitric oxide synthase 2, inducible)	
ID assay	Mm00440502_m1
TaQman probe	GCCTTGTGTCAGCCCTCAGAGTACA
Amplicon length	66
Gapdh (Gliceraldehyde-3 phosphate dehydrogenase)	
ID assay	Mm99999915_g1
TaQman probe	GGTGTGAACGGATTTGGCCGTATTG
Amplicon length:	109

### Statistical analysis

Data are presented as means ± standard error of the mean (S.E.M.) or as medians when appropriate. Student’s *t* test, one-way or two-way analysis of variance followed by Bonferroni test were used to compare means, and Kruskal–Wallis and Dunn’s tests were used to compare medians. Data analyses were performed with GraphPad Prism 6.0 software (GraphPad Software Inc., USA). A *p* value < 0.05 was considered significant.

## Results

### CPT-11 increases the number of mast cells in the small intestine

**Fig. 1:**
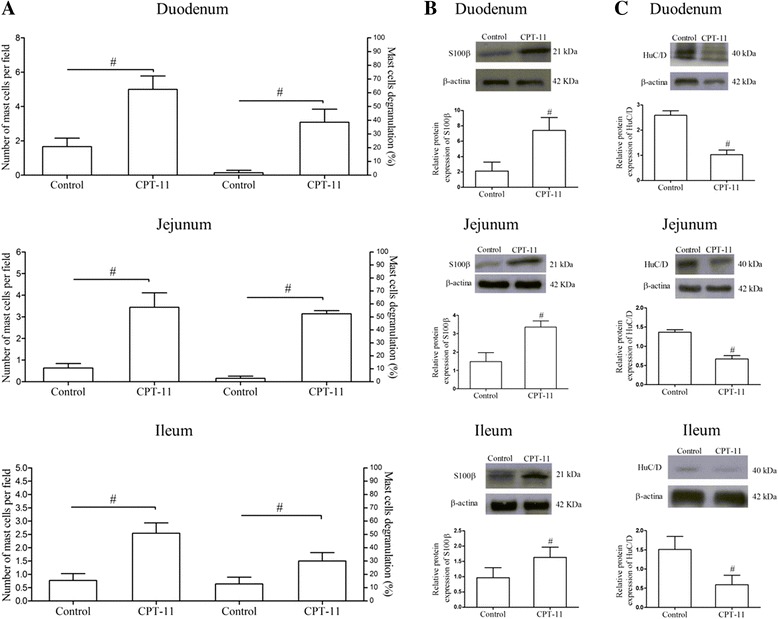
CPT-11 induces mastocytosis and alters S100β and HuC/D protein expression in small intestine. **a** Total and degranulated mast cells were count in the duodenum, jejunum, and ileum in ten microscope field (×400)/slide per mice from six mice in each group. *Bars* represent mean ± SEM of number of mast cell/field or percentage of degranulated mast cell per field. ^#^ *p* < 0.05 versus control group. **b** Representative images of western blot showing S100β and beta-actin (loading control) protein expression. Quantitative analysis indicated that CPT-11 increased S100β protein expression in the small intestine. **c** Representative images of western blot showing HuC/D and beta-actin (loading control) protein expression. Quantitative analysis indicated that CPT-11 decreased HuC/D protein expression in the small intestine. *Bars* represent mean ± SEM for six tissue samples in each group. ^#^ *p* < 0.05 versus control group. Student’s *t* test

Considering that mediators released from activated mast cell are involved in neuron death [22], we investigated whether CPT-11 could increase the number of mast cells in the small intestine and induce their degranulation, using toluidine blue staining. We found that CPT-11 enhanced the total and degranulated mast cell number in the small intestine of mice subjected to CPT-11-induced intestinal mucositis when compared with the control group (Fig. 1a, Additional file 1: Figure S1).

### CPT-11 enhances the expression of S100β and reduces HuC/D in the small intestine

Western blotting for glial (S100β) and neuron (HuC/D) markers was performed to evaluate the effect of CPT-11 on the enteric glial cells and neurons, respectively. CPT-11 enhanced the S100β protein expression (Fig. 1b) and decreased the HuC/D protein expression in the three segments of the small intestine when compared with the control group (Fig. 1c).

### Pre-degranulation of mast cells prevents the CPT-11-induced histological changes

In order to investigate whether the expression of S100β and HuC/D, markers of enteric glia and neuron, respectively, are altered in the ENS of animals treated with CPT-11 and to investigate the role of mast cells in this effect, the mice were previously pretreated with 48/80, a compound that promotes mast cells degranulation, before the first injection of CPT-11 to prevent the mast cell degranulation. The degranulation of mast cells by compound 48/80 was previously confirmed by histological analysis of each small intestine section stained with toluidine blue (data not shown). In addition, mast cells were immunostained using an anti-tryptase antibody. CPT-11 increased (*p* < 0.05) the number of tryptase positive immunostaining cells in the duodenum and jejunum segments, but not in the ileum (*p* > 0.05), when compared with control group (Additional file 2: Figure S2). The pretreatment with compound 48/80 prevented the CPT-11-induced increase in the tryptase expression in both duodenum and jejunum sections (Additional file 2: Figure S2).

**Fig. 2:**
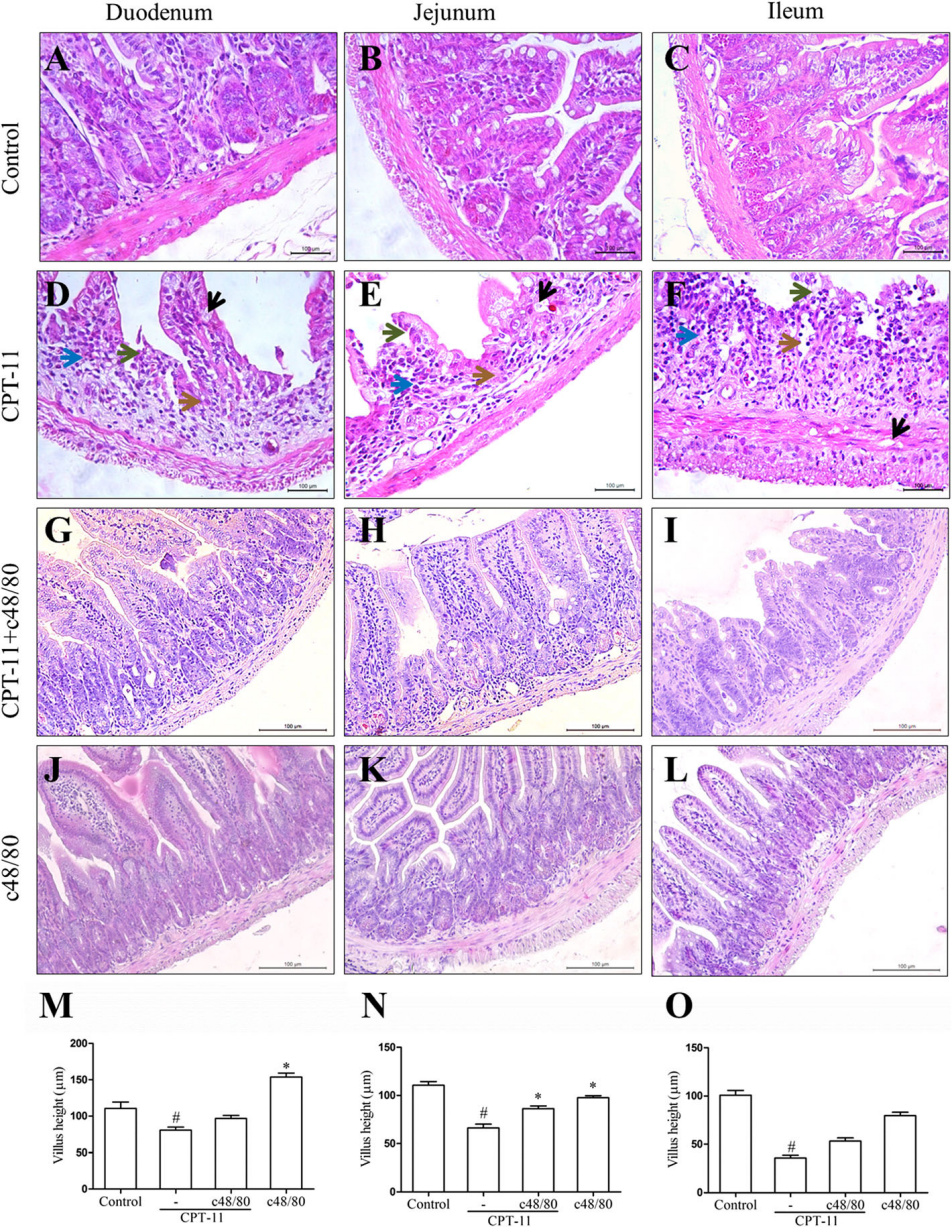
Effects of the mast cells pre-degranulation in the intestinal histopathological analysis and villus height: duodenum, jejunum, and ileum of controls (untreated); animals with CPT-11-induced intestinal mucositis; pretreated with c48/80 and submitted to CPT-11-induced intestinal mucositis or treated only with c48/80. **d** *–* **f** CPT-11 induces shortening of the villus (*green arrows*), loss of crypt architecture (*brown arrows*), intense inflammatory cell infiltrate (*blue arrows*), and cell vacuolization (*black arrows*) in duodenum, jejunum, and ileum. Hematoxylin and eosin; *scale bar* correspond to 100 μm in all figures (**a**–**l**). Segments of duodenum (**m**), jejunum (**n**), and ileum (**o**) were collected for measurement of villus height (ten villus/slide). *Bars* represent mean ± SEM of eight mice in each group. ^#^ *p* < 0.05 versus control group, *p < 0.05 versus CPT-11 group. One-way ANOVA followed by Bonferroni

The histopathology of the small intestine segments (duodenum, jejunum, and ileum) of animals subjected to CPT-11-induced intestinal mucositis showed severe destruction of the crypts, villus shortening (Fig. 2m–o), vacuolization of epithelial and smooth muscle cells, and intense infiltration of inflammatory cells into the mucosa, resulting in high histological scores (Fig. 2d–f; Table 2), when compared with the group not subjected to CPT-11-induced intestinal mucositis (control group; Fig. 2a–c; Table 2). The pretreatment with compound 48/80 was able to prevent the villus atrophy in the jejunum (Fig. 2n), but neither in the duodenum (Fig. 2m) nor in the ileum (Fig. 2o). Compound 48/80 also prevented the destruction of the crypts, and the infiltration of inflammatory cells into the mucosa of the three small intestine segments (Fig. 2g–i), resulting in significant differences in the histological scores (Table 2) between CPT-11 + c48/80 and CPT-11 groups observed in jejunal segments. Compound 48/80 by itself did not cause any damage to the small intestine, as illustrated by representative photomicrographs of duodenum (Fig. 2j), jejunum (Fig. 2k), and ileum (Fig. 2l) of a mouse treated with this compound. No significant differences were found between the c48/80 and the control groups (Table 2).

**Table 2:** Histological scores for intestinal mucositis

Intestine segments	Experimental groups			
	Control	CPT-11	CPT-11 + c48/80	c48/80
Duodenum	0 (0–0)	4 (2–4)#	2 (1–3)#	0 (0–1)*
Jejunum	0 (0–0)	4 (3–4)#	2 (0–4)#*	0 (0–1)*
Ileum	0 (0–0)	4 (2–4)#	4 (3–4)#	0 (0–0)*

Data represent median values (and range) of scores from 0 to 4 according to the presence of necrotic cells, loss of crypt architecture, shortened villi, and hypertrophy/hyperbasophilic in the crypt cells. Data were analyzed by using Kruskal–Wallis and Dunn’s test (*n* = 8). ^#^ *p* < 0.05 versus control group, *p < 0.05 versus CPT-11 group

### Pre-degranulation of mast cells prevents the CPT-11-induced macrophages activation in duodenum and jejunum

**Fig. 3:**
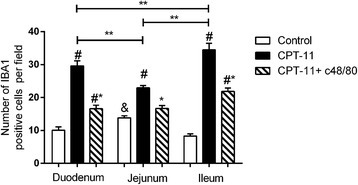
Mast cell pre-degranulation prevents CPT-11-induced increase of Iba1 immunostained cells in the small intestine. *Graph* represents the mean ± SEM of the number of Iba1 positive cells in duodenum, jejunum, and ileum in ten microscope field per section from four mice in each group. *White*, *black*, and *crosshatch bars* represent, respectively, control, CPT-11, and CPT-11 + c48/80 group. ^#^ *p* < 0.05 versus control group. *p < 0.05 versus CPT-11 group. &*p* < 0.05 versus duodenum control group or ileum group control group. **p < 0.05. One-way ANOVA followed by Bonferroni

To further test the role of CPT-11-mediated mast cell degranulation on the macrophages activation, we performed immunohistochemistry to Iba1, an established marker for activated macrophages, in duodenum, jejunum, and ileum sections. Among the three small intestine segments of the control group, jejunum displayed the greatest (*p* < 0.05) number of Iba1 positive immunostaining cells. CPT-11 enhanced (*p* < 0.05) the number of Iba1-positive immunostaining cells in the three small intestine segments, but mostly in the ileum (*p* < 0.05), when compared with control group (Fig. 3). The pretreatment with compound 48/80 prevented the CPT-11-induced increase in the Iba1 expression in the three small intestine segments evaluated. Taken together, these results suggest that CPT-11 induces macrophage activation, and this effect is dependent on the previous release of mast cell mediators (Fig. 3).

### Pre-degranulation of mast cells prevents the CPT-11-induced inflammation and weight loss

**Fig. 4:**
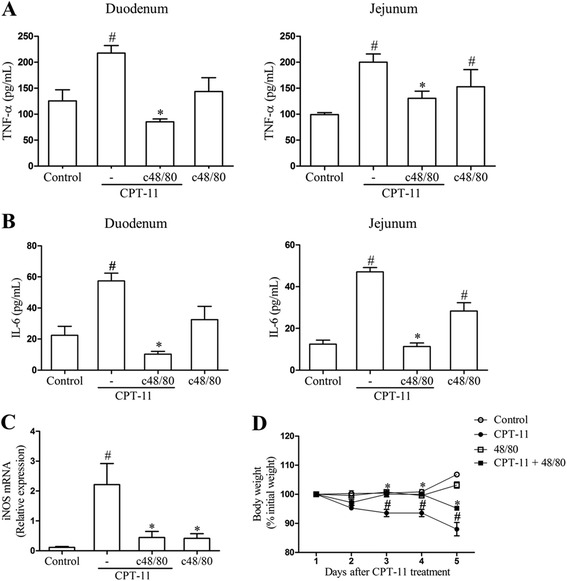
Pre-degranulation of mast cells reduces inflammation in duodenum and jejunum and weight loss of mice submitted to CPT-11-induced intestinal mucositis. **a** Pretreatment with c48/80 diminishes TNF-α levels in duodenum and jejunum of mice submitted to CPT-11-induced intestinal mucositis. **b** Pretreatment with c48/80 diminishes IL-6 levels in duodenum and jejunum of mice submitted to CPT-11-induced intestinal mucositis. **c** Pretreatment with c48/80 decreases iNOS mRNA expression in mice submitted to CPT-11-induced intestinal mucositis. *Bars* represent mean ± SEM of six mice in each group. ^#^ *p* < 0.05 versus control group, *p < 0.05 versus CPT-11 group. One-way ANOVA followed by Bonferroni. **d** Body weight changes are shown as percentages of the baseline value. *Bars* represent mean ± SEM of six mice in each group. ^#^ *p* < 0.05 versus control group, *p < 0.05 versus CPT-11 group. Two-way ANOVA followed by Bonferroni

In order to reinforce that the mast cell pre-degranulation could be able to prevent the CPT-11-induced inflammation, we assessed the levels of TNF-α and IL-6 in the duodenum and jejunum. We found that the pretreatment with compound 48/80 prevented (*p* < 0.05) the increase in both TNF-α (Fig. 4a) and IL-6 (Fig. 4b) levels, induced by CPT-11, since significant differences were found between CPT-11 + c48/80 and CPT-11 groups. Moreover, the pretreatment with compound 48/80 prevented the increase in the iNOS genic expression, induced by CPT-11, in the duodenum (Fig. 4c). It must be noted that the administration of compound 48/80 alone (not followed by CPT-11 administrations) had no effect on the levels of TNF-α, IL-6, or iNOS genic expression in the duodenum, since no statistical differences were detected between c48/80 and control groups. On the other hand, compound 48/80 itself induced an increase in the TNF-α and IL-6 levels in the jejunum compared to the control group.

The pretreatment with compound 48/80 prevented the CPT-11-induced weight loss, while the administration of compound 48/80 alone had no effect on this parameter (Fig. 4d).

### Pre-degranulation of mast cells prevents the CPT-11-induced reactive gliosis and neuron loss

**Fig. 5:**
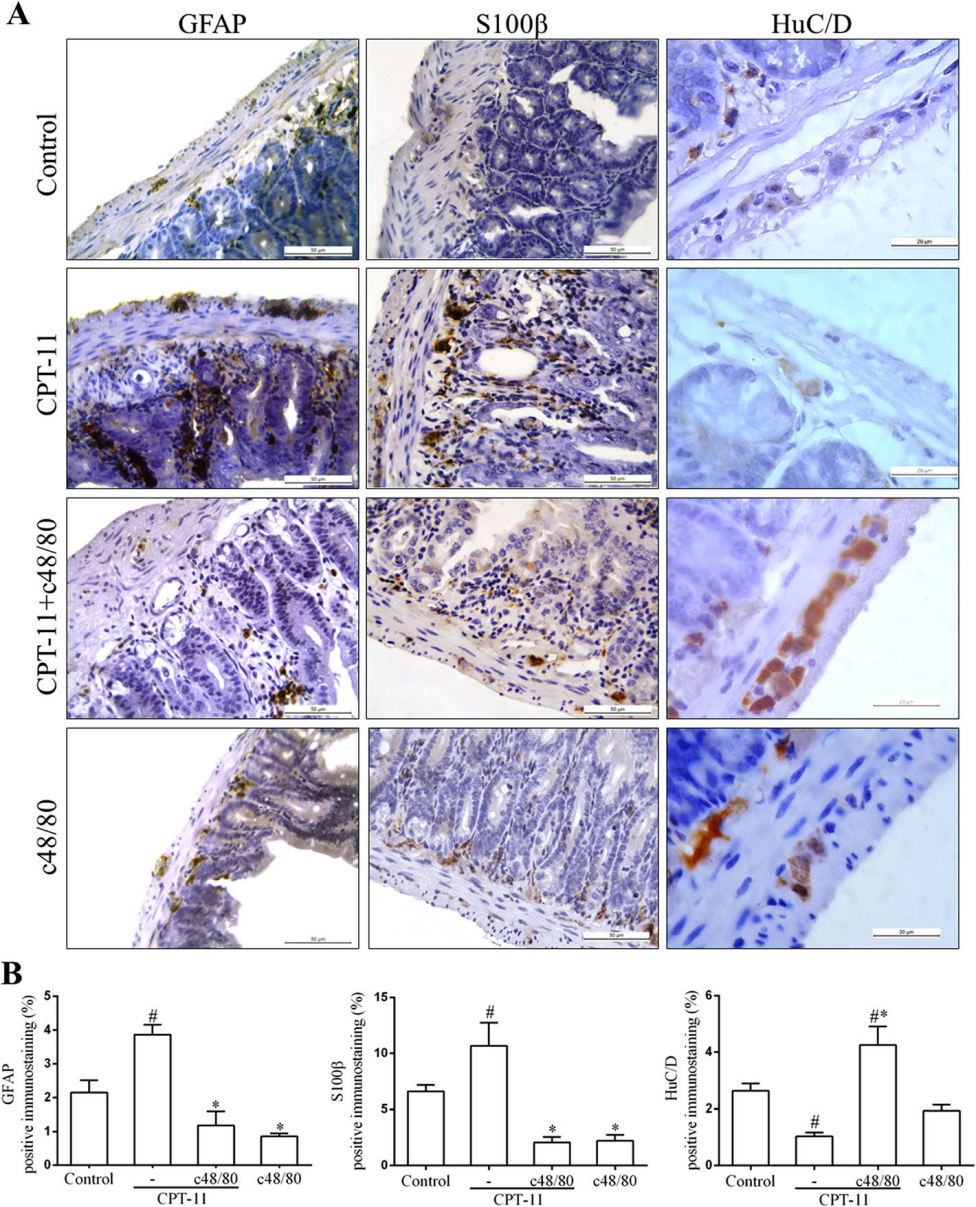
Mast cell pre-degranulation prevents CPT-11-induced increase of GFAP and S100β immunostaining and reduction of HuC/D immunostaining in duodenum of mice. **a** Representative images illustrating GFAP, S100β, and HuC/D immunostaining in the mucosa, submucosa, and myenteric plexuses. *Scale bar* corresponds to 50 μm (GFAP and S100β) or 20 μm (HuC/D). **b** *Graphs* represent the mean ± SEM of the percentage of GFAP, S100β, or HuC/D positive immunostaining in duodenum related to total tissue in five (GFAP and S100β) or ten (HuC/D) microscope field per mice from six mice in each group quantified using Photoshop. ^#^ *p* < 0.05 versus control group. *p < 0.05 versus CPT-11 group. One-way ANOVA followed by Bonferroni

To examine whether the CPT-11-induced reactive gliosis might be mediated by mast cells, GFAP and S100β expressions were investigated by immunohistochemistry in the small intestine sections collected from mice pretreated, or not, with compound 48/80 before the CPT-11 administrations. An increased GFAP immunostaining was clearly observed in mucosa, submucosa, and myenteric plexuses of the duodenum of mice subjected to CPT-11-induced intestinal mucositis (CPT-11 group) when compared with the control group (Fig. 5a). The pretreatment with compound 48/80 (*p* < 0.05) prevented the increase of both GFAP and S100β-positive cells in the duodenum of the CPT-11 + c48/80 group when compared with CPT-11 group (Fig. 5a, b).

To investigate the participation of mast cells in the CPT-11-induced neuron loss, immunohistochemistry to HuC/D was performed in the intestine sections collected from mice pretreated, or not, with compound 48/80. CPT-11 induced a decrease (*p* < 0.05) in the HuC/D expression when compared with the control group. The pretreatment with compound 48/80 was able to prevent (*p* < 0.05) this effect induced by CPT-11. In addition, no statistical differences were detected between c48/80 and control group. Figure 5 shows a considerable reduction in the HuC/D-positive immunostained cells in the duodenum of CPT-11 group when compared with the immunostaining detected in the CPT-11/c48/80 group (Fig. 5a, b).

### Pre-degranulation of mast cells prevents the CPT-11-induced upregulation of S100β and GFAP gene expression and S100β protein expression

**Fig. 6:**
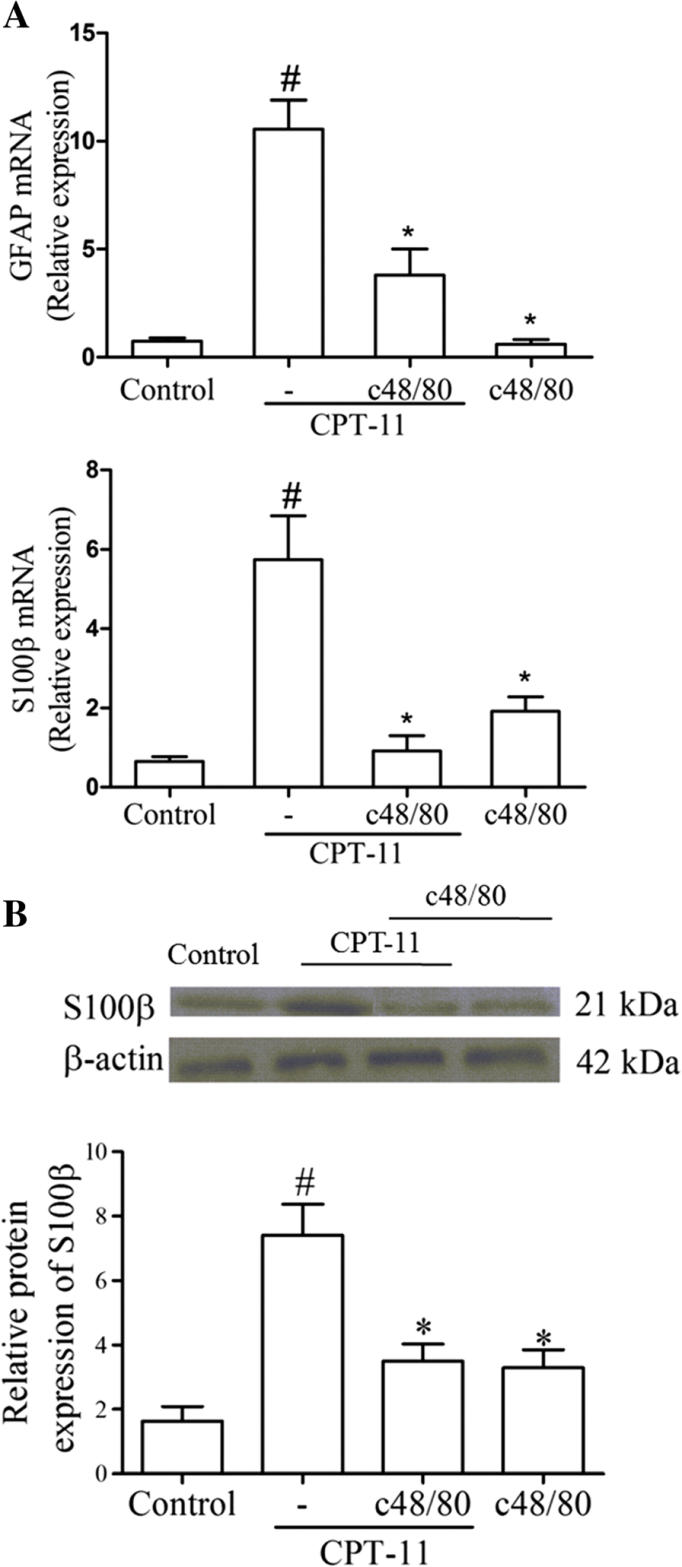
Mast cell pre-degranulation downregulates CPT-11-induced GFAP and S100β RNAm expression and S100β protein expression in duodenum. **a** GFAP mRNA expression and S100β mRNA expression in the duodenum were evaluated by qPCR. **b** S100β protein expression was evaluated by western blot. *Bars* represent mean ± SEM of six mice in each group. ^#^ *p* < 0.05 versus control group, *p < 0.05 versus CPT-11 group. One-way ANOVA followed by Bonferroni

It was observed that a significant increase in the GFAP and S100β genic expression in the duodenum of animals subjected to CPT-11-induced intestinal mucositis (CPT-11 group) compared with control group (Fig. 6a). The pretreatment with compound 48/80 was able to prevent (*p* < 0.05) this effect, since significant differences were found between the CPT-11 and CPT-11/c48/80 groups.

Moreover, it was observed that an increase in the S100β protein expression in the duodenum of mice subjected to CPT-11-induced intestinal mucositis when compared with normal mice (control group). This effect was prevented by the pretreatment with compound 48/80. A significant reduction in the S100β protein expression was observed in the duodenum of CPT-11 + 48/80 group, when compared to the non-pretreated group (CPT-11) (Fig. 6b). The compound 48/80 itself had no effect in the GFAP and S100β expressions since no significant differences were found between c48/80 and control groups (Fig. 6a, b).

### Discussion

In the present study, we provide sufficient evidence to suggest that mast cells activation makes a significant contribution to the pathophysiological processes associated with CPT-11-induced intestinal mucositis through the release of pro-inflammatory mediators and activation of glial cells, detected by increased expression of GFAP and S100β. These mechanisms might have an impact on intestinal dysfunction due to neuronal injury.

The CPT-11-induced intestinal damage is indicated in the current work by shortened villi, loss of crypts architecture, and intense inflammatory cell infiltrate, in accordance with previous studies described in the literature [9, 10, 27, 28]. The participation of neutrophils and macrophages in the pathogenesis of CPT-11-induced intestinal mucositis had been reported [9, 10]. However, as far as we know, the present study shows for the first time the involvement of mast cells in the intestinal side effects of CPT-11, including damage to the ENS. Our data strongly suggests that CPT-11 has the capacity to induce morphological changes in the ENS through the release of mast cells mediators, since we found a significantly enhanced of total and degranulated mast cells in the three small intestine segments of mice subjected to CPT-11-induced intestinal mucositis. Previous studies also documented mastocytosis associated with irritable bowel syndrome [19], Crohn’s disease [18], ulcerative colitis [29], and ischemia-reperfusion injury in animals [30].

In order to investigate the involvement of mast cells in the pathophysiology of CPT-11-induced intestinal mucositis, the mast cells were previously degranulated by pharmacological treatment of the animals with the compound 48/80 [31], which leads to impairment of mast cells function through their degranulation.

Mast cells are normal residents of mucosal tissues, but their number can change markedly during immune responses, infections, and other disorders [32]. It is well established that mast cells, stimulated via the high affinity receptor for immunoglobulin E (IgE) or via any of multiple other mechanisms, can release a diverse spectrum of biologically active mediators, and such products can have many different effects on immune or structural cells present in mucosal tissues [32, 33]. These pre-stored mediators include vasoactive amines, neutral proteases, and some preformed cytokines, including TNF-α. Mast cells are also able to synthesized and secrete a large number of chemokines, growth factors, and cytokines, including TNF-α, IL-1β, IL-6, and many others [32]. On the other hand, inflammatory cytokines can stimulate mast cell degranulation during inflammatory conditions, where they are activated by non-allergic triggers [34]. It has been cited that IL-18 and IL-33, cytokines established involved in the pathogenesis of intestinal mucositis, may directly activate mast cells [35, 36].

Mast cell production of chemotactic factors can enhance the recruitment of multiple inflammatory cells, resulting in amplification of the inflammatory reaction. The mast cell-mediated enhancement of inflammation could induce damage of host tissues. Accordingly, we detected that overall intestinal architecture and villi height were noticeably preserved in the group pretreated with compound 48/80, associated with decreased levels of TNF-α, IL-6, and iNOS gene expression, whose role in the chemotherapy-induced intestinal mucositis had been previously confirmed [8, 11, 27]. Consistently, the pre-degranulation of mast cells by compound 48/80 in models of lethal sepsis resulted in a significant decrease in the serum levels of TNF-α and interleukin-8 (IL-8) [24]. Moreover, we demonstrated that CPT-11 activates macrophages in the three small intestine segments, as seen by immunohistochemistry. Our results also suggest that the macrophage activation is associated with mast cell mediators, especially in duodenum and jejunum. These data may be a possible explanation to the lower effect of the compound 48/80 on the CPT-11-induced inflammatory response in ileum in comparison with duodenum and jejunum segments. The differences among the small intestine portions, regarding the population of immune cells, the intestinal microbiota, and the size of the intestinal villi have been described in the literature. Villi are longer in duodenum and jejunum, where most digestion occurs. Ileum, on the other hand, has markedly shorter villi and lower levels of brush border enzymes [37]. The accumulation of proinflammatory macrophages has also been detected in the colon of animals and patients with inflammatory bowel disease and has been linked to disease severity and progression [38]. Taken together, our findings suggest that mast cells and macrophages might be important sources of cytokines and enzymes that contribute to gastrointestinal injuries observed in the CPT-11-induced intestinal mucositis and subsequent loss of body mass. Further studies are needed to assess the influence of intestinal mast cells mediators on migration and/or activation of other inflammatory cells, such as neutrophils. Cytokines have been reported to stimulate intestinal secretion and motility [20, 21] to cause diarrhea and intestinal dysfunction, which are key signs and symptoms of intestinal mucositis [8, 9]. It is well established that proinflammatory cytokines, such as IL-6 and TNF-α, are potent inducers of iNOS in a wide variety of cell types, with consequent production of nitric oxide (NO). We previously demonstrated that chemotherapy-induced iNOS activation plays a critical role in the intestinal mucosal injury [8, 39]. The participation of NO in the present study is supported by the increase in the iNOS gene expression in the intestinal tissue, induced by CPT-11 administrations.

Based on previous studies demonstrating that CPT-11 causes intestinal over-contractility [8, 9], which might be dependent on cytokines and glial cells, we decided to investigate the effect of CPT-11 on the ENS, which autonomously controls gastrointestinal motility, secretion, and blood flow [40]. We found that CPT-11 leads to a pronounced loss of neurons in the three small intestine segments (jejunum and ileum data not shown). These findings are supported by the increased expression of GFAP and S100β, two established markers for glia cells, associated with a reduced positivity for HuC/D, a neuronal marker, in the small intestine of mice subjected to CPT-11-induced mucositis (jejunum and ileum data not shown), suggesting glial activation and neuronal loss in the ENS. In accordance with our data, several inflammatory intestinal diseases, such as celiac [12], Crohn’s diseases [13], and ulcerative colitis [14, 15] had been associated with significant enteric nervous system alterations.

GFAP, the major protein constituent of glial intermediate filaments in the differentiated fibrous and protoplasmic astrocytes of the central nervous system, is involved in controlling the shape and the movement of these cells [41, 42]. Increased expression of GFAP and S100β represents astroglial activation and gliosis [41, 43]. In fact, under normal basal conditions, only subsets of intestinal enteric glia express GFAP, and most of the enteric glia cells coexpress S100β, proteolipid protein 1 (PLP1), and SRY-related HMG-box10 (Sox10) [44]. Low levels (nanomolar concentrations) of S100β generally stimulate neuronal pro-survival responses [45]. High levels (micromolar concentrations), however, promote pro-apoptotic effects [45] and receptor for advanced-glycation end products (RAGE)-nuclear factor kappa B (NFκB)-dependent release of IL-6 and TNF-α [46, 47]. Given this, the enteric neuronal loss, detected by the decreased expression of HuC/D in the small intestine of mice subjected to CPT-11-induced intestinal mucositis, may be associated with the increase in the S100β expression and subsequent release of proinflammatory cytokines and NO. Accordingly, the literature describes the production of NO by peritoneal rats macrophages stimulated with S100β, in a concentration-dependent manner [48]. In addition, it has been demonstrated that inducers of NO also induce the expression of GFAP, through NO. The NO-coupled guanylate cyclase-cyclic guanosine monophosphate (cGMP)-activated protein kinase pathway seems to be important in the GFAP activation [41], suggesting that the upregulation of GFAP expression in the ENS, induced by CPT-11 in the current work, may follow the NO production.

The effects of CPT-11 in the ENS, as observed in the present work, are at least partially related to mast cell stimulation and degranulation, since decreased expression of GPAF and S100β was observed when compound 48/80 was administered to CPT-11-treated mice, probably as a consequence of its protective effects on the small intestinal mucosa and submucosa during the CPT-11-induced intestinal mucositis, illustrated by lower levels of inflammatory markers release associated with reduced number of degranulated mast cells in the three small intestine segments (jejunum and ileum data not shown). A direct effect of mast cell mediators on the ENS, however, should not be ruled out. Mast cells are suggested to be key elements in the neuro-immune interaction due to their close localization to enteric neurons, vagal nervous fibers, and spinal sensory nerves [21, 49]. In primary cultures of neurons, it was showed that tryptase, a mast cell protease, led to neuronal death via activation of protease-activated receptor-2 (PAR-2) [22]. Furthermore, histamine, lipid mediators, cytokines, and adhesion molecules act through paracrine pathways on the enteric nervous system networks, which in turn regulate mast cell function [50]. Therefore, we speculate that mast cells could account for neuronal death during mucositis likely through similar mechanisms.

## Conclusions

In summary, our results highlight the potential role of mast cells during the inflammatory scenario induced by CPT-11 in the small intestine. These cells seem to release inflammatory mediators that regulate glial cell and macrophages activation, leading to intestinal mucosa damage and reduction of enteric neurons. However, more studies are needed to work out how specifically the activation of mast cells after exposure to CPT-11 leads to GI toxicity, and whether or not mast cells play a direct role in the observed modification of the ENS. In vitro studies are extremely important for that investigation. In addition, it should be noted that, although mouse models are powerful and effective to investigate the pathophysiology of many diseases, their use is not directly applicable for the human. Further studies are needed to propose a therapeutic strategy based on the data presented in this study.

## Additional files

Additional file 1: CPT-11 increases the number of mast cells in the small intestine. Intestinal segments (duodenum, jejunum or ileum) were stained with toluidine blue. Mast cells (black arrows) were counted in all intestinal segments. Scale bar = 50 μm. (TIF 3590 kb)

Additional file 2: Mast cell pre-degranulation prevents CPT-11-induced increase of tryptase immunostainedcells in the duodenum and jejunum of mice. Graph represents the mean ± SEM of the number of tryptasepositive cells in the duodenum, and jejunum in ten microsc opic field per section from four mice in eachgroup. White, black and crosshatch bars represent, respectively control, CPT-11 and CPT-11+c48/80 group.#*P* < 0.05 versus control group. *P < 0.05 versus CPT-11 group. One-way ANOVA followed by Bonferroni. (DOC 70 kb)

## Abbreviations

cDNA: Complementary DNA
cGMP: Cyclic guanosine monophosphate
CPT-11: Irinotecan
DAB: Diaminobenzidine
DNA: Deoxyribose nucleic acid
EGC: Enteric glial cell
ENS: Enteric nervous system
Gapdh: Glyceraldehyde-3-phosphate dehydrogenase
GFAP: Glial fibrillary acidic protein
Iba-1: Ionized calcium-binding adapter molecule
IgE: Immunoglobulin E
IL-18: Interleukin-18
IL-1β: Interleukin-1β
IL-33: Interleukin-33
IL-6: Interleukin-6
IL-8: Interleukin-8
iNOS: Inducible nitric oxide synthase
mRNA: Messenger RNA
NFκB: Nuclear factor kappa B
NO: Nitric oxide
PAR-2: Protease-activated receptor 2
PBS: Phosphate-buffered saline
PCR: Polymerase chain reaction
PGD2: Prostaglandin D2
PLP1: Proteolipid protein 1
RAGE: Receptor for advanced-glycation end products
RNA: Ribonucleic acid
SOX10: SRY-related HMG-box10
TNF-α: Tumor necrosis factor-α
