# Supplementary figures and images for: The involvement of mast cells in the irinotecan-induced enteric neurons loss and reactive gliosis
Source: J Neuroinflammation. 2017 Apr 7;14:79. doi: 10.1186/s12974-017-0854-1 (PMC5384042; doi:10.1186/s12974-017-0854-1)

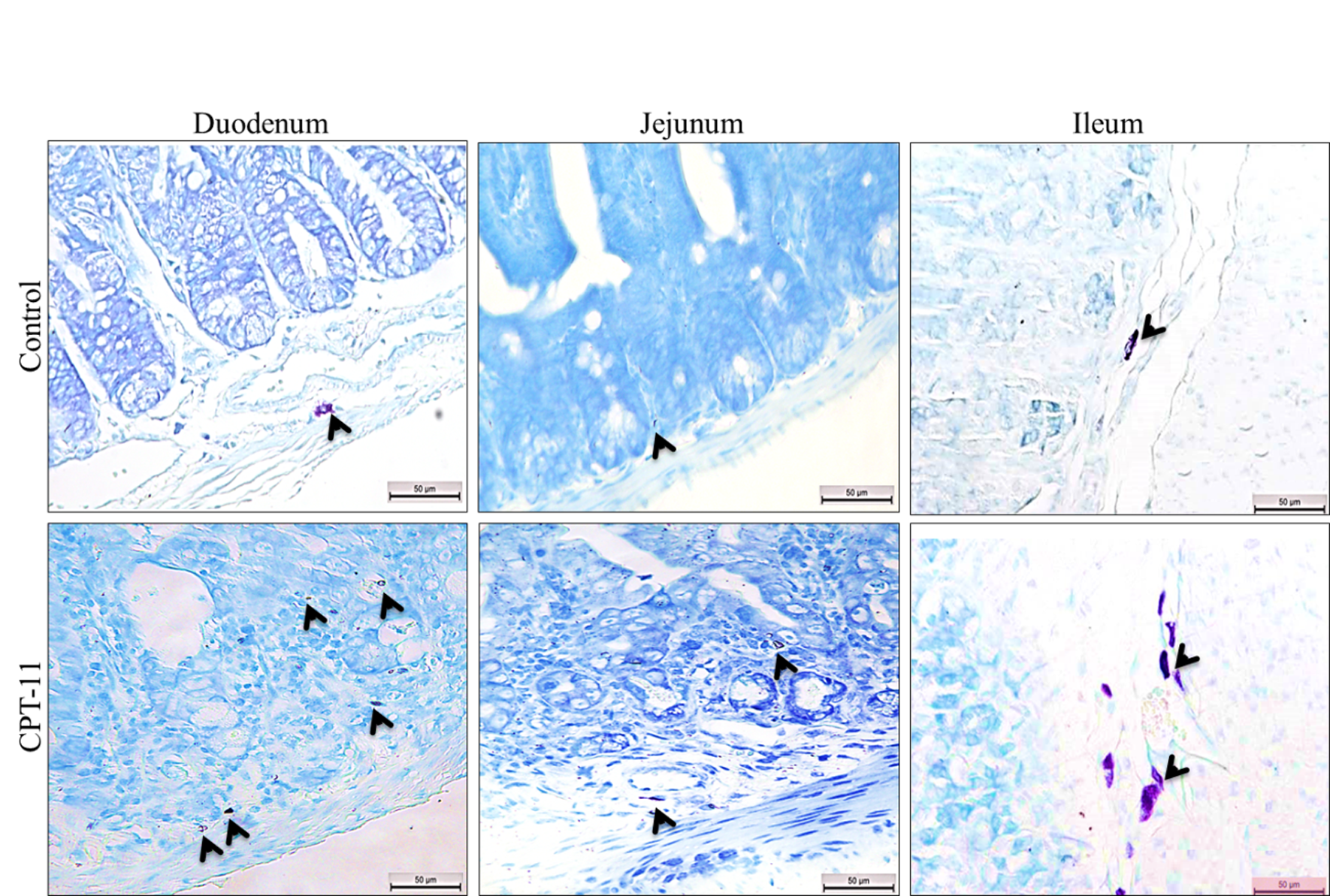

Supplement: Supplementary file 1 — CPT-11 increases the number of mast cells in the small intestine. Intestinal segments (duodenum, jejunum or ileum) were stained with toluidine blue. Mast cells (black arrows) were counted in all intestinal segments. Scale bar = 50 μm. (TIF 3590 kb) [file 12974_2017_854_MOESM1_ESM.tif]
