# Supplementary material for: The involvement of mast cells in the irinotecan-induced enteric neurons loss and reactive gliosis
Source: J Neuroinflammation. 2017 Apr 7;14:79. doi: 10.1186/s12974-017-0854-1 (PMC5384042; doi:10.1186/s12974-017-0854-1)
Supplement: Supplementary file 2 — Mast cell pre-degranulation prevents CPT-11-induced increase of tryptase immunostainedcells in the duodenum and jejunum of mice. Graph represents the mean ± SEM of the number of tryptasepositive cells in the duodenum, and jejunum in ten microsc opic field per section from four mice in eachgroup. White, black and crosshatch bars represent, respectively control, CPT-11 and CPT-11+c48/80 group.#P < 0.05 versus control group. *P < 0.05 versus CPT-11 group. One-way ANOVA followed by Bonferroni. (DOC 70 kb) [file 12974_2017_854_MOESM2_ESM.doc]

**Supplementary 2**

**
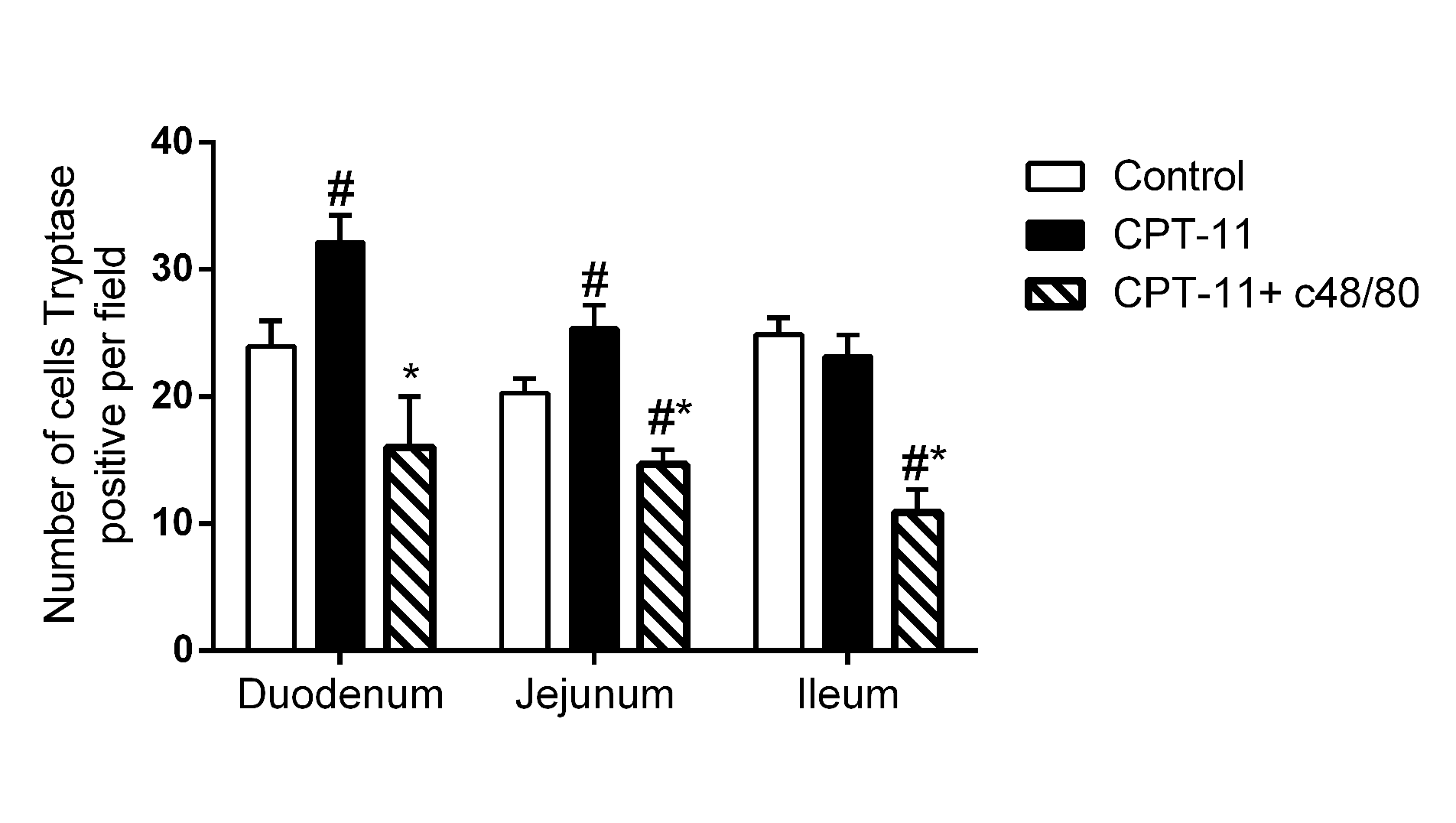
**

Mast cell depletion prevents CPT-11-induced increase of tryptase immunostained cells in duodenum and jejunum of mice. Graph represents the mean ± SEM of the number of tryptase positive cells in duodenum, and jejunum in ten microscope field per section from four mice in each group. White, black and crosshatch bars represent respectively control, CPT-11 and CPT-11+c48/80 group. # P< 0.05 versus control group. *P< 0.05 versus CPT-11 group. One-way ANOVA followed by Bonferroni.
